# Incentive payments to general practitioners aimed at increasing opportunistic testing of young women for chlamydia: a pilot cluster randomised controlled trial

**DOI:** 10.1186/1471-2458-10-70

**Published:** 2010-02-17

**Authors:** Jade E Bilardi, Christopher K Fairley, Meredith J Temple-Smith, Marie V Pirotta, Kathleen M McNamee, Siobhan Bourke, Lyle C Gurrin, Margaret Hellard, Lena A Sanci, Michelle J Wills, Jennifer Walker, Marcus Y Chen, Jane S Hocking

**Affiliations:** 1 Sexual Health Unit, Melbourne School of Population Health, The University of Melbourne, Carlton, Victoria 3053, Australia; 2 Melbourne Sexual Health Centre, Alfred Health, Carlton, Victoria 3053, Australia; 3 Primary Care Research Unit, Department of General Practice, The University of Melbourne, Carlton, Victoria 3053, Australia; 4 Family Planning Victoria, Box Hill, Victoria 3128, Australia; 5 Centre for Molecular, Environmental, Genetic and Analytic Epidemiology, Melbourne School of Population Health, The University of Melbourne, Carlton, Victoria 3053, Australia; 6 Centre for Population Health, Burnet Institute, Melbourne, Victoria 3004, Australia; 7 General Practice Divisions Victoria, 458 Swanston Street, Carlton, Victoria 3053, Australia; 8 Centre for Women's Health, Gender and Society, Melbourne School of Population Health, The University of Melbourne, Carlton, Victoria 3053, Australia

## Abstract

**Background:**

Financial incentives have been used for many years internationally to improve quality of care in general practice. The aim of this pilot study was to determine if offering general practitioners (GP) a small incentive payment per test would increase chlamydia testing in women aged 16 to 24 years, attending general practice.

**Methods:**

General practice clinics (n = 12) across Victoria, Australia, were cluster randomized to receive either a $AUD5 payment per chlamydia test or no payment for testing 16 to 24 year old women for chlamydia. Data were collected on the number of chlamydia tests and patient consultations undertaken by each GP over two time periods: 12 month pre-trial and 6 month trial period. The impact of the intervention was assessed using a mixed effects logistic regression model, accommodating for clustering at GP level.

**Results:**

Testing increased from 6.2% (95% CI: 4.2, 8.4) to 8.8% (95% CI: 4.8, 13.0) (p = 0.1) in the control group and from 11.5% (95% CI: 4.6, 18.5) to 13.4% (95% CI: 9.5, 17.5) (p = 0.4) in the intervention group. Overall, the intervention did not result in a significant increase in chlamydia testing in general practice. The odds ratio for an increase in testing in the intervention group compared to the control group was 0.9 (95% CI: 0.6, 1.2). Major barriers to increased chlamydia testing reported by GPs included a lack of time, difficulty in remembering to offer testing and a lack of patient awareness around testing.

**Conclusions:**

A small financial incentive alone did not increase chlamydia testing among young women attending general practice. It is possible small incentive payments in conjunction with reminder and feedback systems may be effective, as may higher financial incentive payments. Further research is required to determine if financial incentives can increase testing in Australian general practice, the type and level of financial scheme required and whether incentives needs to be part of a multi-faceted package.

**Trial Registration:**

Australian New Zealand Clinical Trial Registry ACTRN12608000499381.

## Background

*Chlamydia trachomatis *is the most commonly notified bacterial sexually transmitted infection (STI) in Australia, with notification rates rising nearly fourfold over the past ten years, from 73.5 per 100,000 in 1999 to 273.8 per 100,000 in 2008 [1]. Left untreated, chlamydia can lead to serious reproductive sequelae including pelvic inflammatory disease, ectopic pregnancy and infertility [2,3]. Given that up to 90% of infections are asymptomatic [3], increased testing is required to effectively detect and control chlamydia infection and limit its associated morbidity.

In Australia, national chlamydia testing guidelines issued by the Royal Australian College of General Practitioners (RACGP) [4] recommend annual testing of sexually active young people under the age of 25 years. Australian data show that diagnosis rates are highest among young women aged under 25 years, who accounted for just over 40% of all chlamydia notifications in 2008 [1].

Given that almost 90% of women in this age group consult a general practitioner (GP) at least once a year [5], general practice is considered an ideal site through which to offer chlamydia testing. Currently in Australia only 12% of women in this age group are tested for chlamydia each year [6]. Mathematical modelling has suggested that testing needs to increase to approximately 30% annually among men and women in this age group to achieve a significant reduction in prevalence among women [7].

Previous randomised controlled trials (RCT) have used a variety of interventions to increase chlamydia testing in general practice, including educational or continuing medical education interventions [8,9], computerised reminders [10] and concurrent pap smear and chlamydia testing [11], however all have had limited success in increasing testing. In practice, a number of barriers to increased testing exist for GPs including: a lack of time, insufficient knowledge about the benefits of testing, discomfort raising the issue of sexual health in unrelated consultations and difficulty remembering to offer testing to patients [12-15].

Financial incentives have been used for many years internationally to improve quality of care in general practice [16-18]. In the UK, as part of the pilot chlamydia testing program, opportunistic chlamydia testing for young women increased when general practices were offered financial incentives for testing [19]. In Australia, the use of GP financial incentives was first introduced in 1998 in the form of the General Practice Immunisation Incentive Scheme (GPII) and successfully increased childhood immunisation to target rates [5]. The use of GP financial incentives to increase chlamydia testing in Australian general practice has been recommended by a number of researchers in the field [5,10,12,15], however to date has not been examined.

The aim of this pilot study was to use a RCT design to determine if offering a $AUD5 payment per chlamydia test to GPs would increase chlamydia testing among 16 to 24 year old women attending general practice.

## Methods

### Setting

A cluster RCT was undertaken in general practice clinics in the State of Victoria (population 5.4 million) [20], Australia, between May 2008 and January 2009.

### Eligibility

General practices were eligible to participate if they were located within Victoria, had a minimum of two full time equivalent GPs willing to participate and collectively saw a minimum of 250 women aged 16 to 24 years in the 12 months prior to the trial.

### Recruitment methods

General practices were selected from a database collated from the Victorian 'Yellow Pages' telephone directory. In order to provide adequate representation of general practices across the state, practices were recruited by geographical location using the Accessibility Remoteness Index of Australia (ARIA) [21] and socio-economic status using the Socio-Economic Indexes for Areas (SEIFA) [22]. A total of eight practices were recruited in metropolitan areas (highly accessible) - two in each SEIFA quartile - and four in regional/rural areas (accessible/moderately accessible).

Practices were initially telephoned by a research assistant to invite participation and provide further information to interested and eligible clinics. Interested practices were visited by a research assistant to further outline the study and obtain consent from GPs. GPs consented to the collection of de-identified chlamydia testing data and consultation data on female patients 16 to 24 years old. A total of 145 practices were approached to recruit 12 practices into the study. Reasons for non-participation included a lack of response from GPs (57%), no interest from GPs (33%), a lack of time (4%), interest shown after recruitment period had ceased (4%) and clinic participation in other studies (2%).

### Randomisation

The unit of randomisation was the general practice. The eight metropolitan practices were paired within the four SEIFA quartiles and one practice in each quartile was randomised to receive the intervention protocol and the other the control protocol, using a pre-determined randomisation sequence prepared by the trial statistician. The statistician was contacted to make the randomised assignment once two practices had been recruited for any particular SEIFA quartile. The four regional/rural practices were also paired - two rural and two regional - and were assigned within pairs to intervention and control in the same way as the metropolitan practices. The allocation was not revealed to staff at either of the paired practices until representatives from both had completed the pre-trial requirements (questionnaire, audit, education session). GPs in the practice were then contacted by letter to inform them of the allocation relevant to their practice and reminded of testing payment or non-payment.

### Intervention and control

GPs in both the intervention and control groups were required to complete a pre-trial questionnaire, a clinical audit and an education session prior to the commencement of the trial. The self-completed pre-trial questionnaire collected information about GPs characteristics, knowledge, attitudes and practices regarding chlamydia testing and was conducted both pre and post trial by all participating GPs. A clinical audit was undertaken at each practice to collect details about issues likely to have an impact on chlamydia testing in that clinic. Audit data were used to develop an ideal individualized chlamydia testing pathway for each clinic, which incorporated current best practice for testing in the primary care setting of annual chlamydia testing for sexually active women aged 16 to 24 years [23]. GPs were advised to collect specimens for testing by first pass urine, self-collected vaginal swab or endocervical swab. Participating GPs were eligible to enroll in related chlamydia education activities accredited under the RACGP Quality Assurance and Continuing Professional Development Program (QA&CPD) [24].

Following the audit, an education session was held at each practice to further inform GPs about chlamydia testing, management of test results and methods of introducing the subject of testing to patients. Practices were provided with waiting room chlamydia posters, pamphlets and chlamydia screening flow charts. A DVD recording of the education session was available for doctors unable to attend. At the request of GPs, tear off pads with brief information sheets for patients specifically about the reasons for testing and the simplicity of testing and treatment were produced and distributed to the practices.

Mid trial, GPs in the intervention group received a letter to remind them of the incentive offered for chlamydia testing. They were not provided with any information about the number of tests performed to date nor the amount of money they had accrued through testing. Payment was made to GPs at the end of the trial period. All practices received an honorarium amount of $AUD1000 in recognition of GPs time spent out of usual roles in participating in the trial.

### Outcome measures and data collection

The primary study outcome was the difference in the proportion of women tested for chlamydia in the control and intervention groups before and during the intervention. Consultation and chlamydia testing data were collected for two time periods: 12 months prior to and 6 months of the intervention. Data were not collected on the number, characteristics or identities of women who were offered or declined testing during the trial.

With the permission of all participating GPs, pathology laboratories were provided with each GPs unique provider number, specific to their place of practice. The total number of women aged 16 to 24 years tested for chlamydia at least once in each study time period was ascertained for each GP as was the total number of women who had at least one positive chlamydia test result. Consultation data were collected from each practice's computerised patient record system on the number of individual women aged 16 to 24 years each GP consulted in the two time periods.

### Sample size

At trial commencement, approximately 6% of Australian women aged 16 to 24 years were being tested each year for chlamydia by GPs [25]. We hypothesized that the rate of individual women tested for chlamydia would rise to 30% in the intervention group (an increase of 24%) and 15% in the control group (change of 9%). Assuming a conservative intracluster correlation of 0.05 for a cluster size of 100 (which assumes a design effect of 6), a total of 12 practices (six in each group) would be required to give 80% power to detect the hypothesized difference with a type 1 error of 5%.

### Statistical analysis

In both groups, the proportion of 16 to 24 year old women tested at least once for chlamydia was estimated using the observed proportion calculated separately for the twelve months prior and six months of the trial. The observed proportion numerator was the number of women tested at least once for chlamydia and the denominator, the individual number of women consulted during the time period. The chlamydia positivity was also calculated and defined as the proportion of patients tested with at least one positive test. Confidence intervals for all proportions (using the binomial distribution) and a p-value for the comparison of estimated proportions between study groups (using the chi-squared test) separately for the 12 month and 6 month periods were calculated, adjusted for clustering by GP.

A mixed effects logistic regression model, with two level hierarchy (patient and individual GP) was fitted to the data including results from both the intervention and control group for both time periods. The model included fixed regression effects for GP age, GP gender, whether the GP was aware of the RACGP chlamydia testing guidelines (RACGP 'Red Book'), whether the GP had postgraduate qualifications and for an interaction between the intervention group and the time period.

Statistical analysis was performed using Stata statistical software 10.0 [26]. Questionnaire data were analyzed using Statistical Package for the Social Sciences (SPSS) version 17.0 [27].

### Ethical approval

Ethical approval for this study was granted by the human research ethics committee of The University of Melbourne (HREC No: 050747).

### Trial registration

This study was registered on the Australian New Zealand Clinical Trial Registry, registration number ACTRN12608000499381.

## Results

### Intervention and control groups

**Figure 1:**
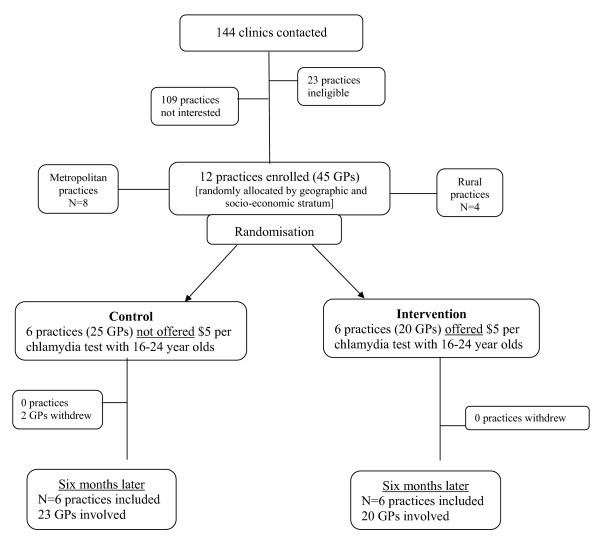
**Flow of participating practices through the trial**. The following figure provides the flow of participating general practices through the trial.

A total of 145 practices were approached to participate in the trial - 26 were ineligible to participate, 107 declined and 12 consented to participate (Figure 1). The first practice was recruited on 12^th ^May 2008, with the final practice completing the 6 month trial on the 3^rd ^January 2009.

Twenty five GPs in the control group and 20 in the intervention group consented to the study. During the trial period, two GPs from one practice (control group) withdrew.

Table 1 outlines the demographic characteristics of participating GPs. Groups were comparable with the exception that control group GPs were more likely to be younger (under 45 years) and have a post-graduate qualification.

**Table 1:** Demographic characteristics of GPs at trial entry (*n *= 43)^#^

Characteristic	Intervention		Control	
	N (%)	(95%CI)	N (%)	(95%CI)
Number of GPs (N)	20		23	
Sex				
Male	9 (45)	(25,67)	11 (48)	(28,68)
Female	11 (55)	(33,75)	12 (52)	(32,72)
Age				
<35 years	3 (15)	(5,38)	2 (9)	(2,30)
35-44 years	0 (0)	(0,17)	9 (39)	(21,60)
45-54 years	13 (65)	(42,83)	8 (35)	(18,56)
55+ years	4 (20)	(8,43)	4 (17)	(7,39)
Sessions worked each week				
<6 sessions	9 (45)	(25,67)	7 (30)	(15,52)
6-10 sessions	10 (50)	(29,71)	16 (70)	(48,85)
11+ sessions	1 (5)	(0,29)	0 (0)	(0,15)
Years in general practice				
0-10 years	4 (20)	(8,43)	6 (26)	(12,48)
11-20 years	6 (30)	(14,53)	9 (39)	(21,60)
21+ years	10 (50)	(29,71)	8 (35)	(18,56)
GPs practice location by ARIA^§^				
Regional/rural	4 (20)	(8,43)	5 (22)	(9,43)
Metropolitan	16 (80)	(57,92)	18 (78)	(57,91)
Postgraduate qualifications				
No	8 (40)	(21,63)	0 (0)	(0,15)
Yes	12 (60)	(37,79)	23 (100.0)	(85,100)
Interest in STIs				
Not interested	2 (10)	(2,33)	2 (9)	(2,30)
Interested	18 (90)	(67,98)	21 (91)	(70,98)
Aware of RACGP 'Red Book'^				
No	2 (10)	(2,33)	2 (9)	(2,30)
Yes	5* (23)	(10,45)	17 (77)	(55,90)

^# ^Adjusted for clustering at GP level

* One missing value

^RACGP 'Guidelines for preventative activities in general practice 6^th ^edition'

^§ ^Accessibility Remoteness Index of Australia - indexes of remoteness in Australia.

### Proportion of patients tested

In the 12 month pre-trial period, 6.2% (95% CI: 4.2, 8.4) of women in the control group were tested for chlamydia compared with 11.5% (95% CI: 4.6, 18.5) in the intervention group (Table 2). The median number of women tested in the control group in the pre-trial period was 6 (range: 0-29) and in the intervention group 8.5 (range 0-133). At this time, GPs in the intervention group were more than twice as likely to be testing women for chlamydia (OR: 2.2; 95% CI: 1.1, 4.4; p = 0.03). During the trial period, there was a non significant increase in testing in both the control group, 6.2% (95% CI: 4.2, 8.4) to 8.8% (95% CI: 4.8, 13.0) (p= 0.1), and in the intervention group, 11.5% (95% CI: 4.6, 18.5) to 13.4% (95% CI: 9.5, 17.5) (p = 0.4) (Table 2). The median number of women tested by GPs in the control group in the trial period was 5 (range: 0-32) and in the intervention group 8 (range: 0-49). The proportion of women who tested positive for chlamydia did not change in the control group but decreased in the intervention group (p = 0.01) during the two time periods (Table 2).

**Table 2:** Pre trial and during trial chlamydia testing numbers, percentages and positivity rates, by control (n = 23) and intervention (n = 20) groups

	Pre-trial period					Trial period						
	**Women consulted** **N**	**Women tested** **N (%)**		**Positive women** **N (%)**		**Women consulted** **N**	**Women tested** **N (%)**		**Positive women** **N (%)**		Women tested* P value	Positive women** P value

**Control** 16-24 yrs	2689	168	(6.2)	16	(9.5)	1792	157	(8.8)	15	(9.6)	p<0.01	p = 0.99
**Intervention** 16-24 yrs	2662	305	(11.5)	28	(9.2)	1589	213	(13.4)	7	(3.3)	p = 0.06	p = 0.01

* The difference in proportion of the number of women tested for chlamydia in the pre-trial and trial periods.

** The difference in proportion of the number of women who tested positive for chlamydia in the pre-trial and trial periods.

The interaction between the estimated effect of time period and intervention group showed that there was no difference in the proportion of women tested between the intervention and control group during the trial (OR = 0.9; 95% CI: 0.6,1.2) (Table 3).

**Table 3:** Adjusted odds ratios (AOR) for the effect of the intervention on the proportion tested.

Variable		AOR (95%CI)
GP gender	Female GPs	1.0
	Male GPs	0.6 (0.4, 1.0)

Age group		
	≤ 44 years	1.0
	≥ 45 years	0.5 (0.3, 0.9)

Postgraduate qualifications		
	No	1.0
	Yes	0.7 (0.4, 1.4)

Knowledge of RACGP 'Red Book' guidelines		
	No	1.0
	Yes	1.2 (0.7, 2.3)

Study group		
	Control	1.0
	Intervention	2.0 (1.1, 3.5)

Time period		
	Pre-trial period	1.0
	Trial period	1.5 (1.2, 1.9)

Interaction of time with intervention		
	Pre-trial period	1.0
	Trial period	0.9 (0.6, 1.2)

# Adjusted for clustering at GP level

### Barriers and facilitators to testing

The most common barriers to increased chlamydia testing identified in GPs' post trial questionnaire were: lack of time (29/43, 69%), difficulty remembering to suggest testing to patients (9/43, 21.4%) and patients' lack of education and awareness about chlamydia testing (9/43, 21.4%). Anecdotally, many GPs noted that while they initially increased chlamydia testing at the beginning of the trial, as time progressed, they no longer remembered to offer testing, and/or forgot about the $5 incentive payment. The three main facilitators to increased testing identified by GPs were: financial incentives for GPs (17/42, 41%), patient education or awareness about testing (13/42, 31%) and computer prompts or reminders to test (11/42, 26%).

## Discussion

In this study we found that offering GPs a $AUD5 testing payment did not increase testing for chlamydia. This was the first RCT to test incentive payments for chlamydia testing. Systematic reviews of RCT's assessing the effectiveness of financial incentives to improve health care quality are limited, and often show mixed results [17,18,28]. Observational studies have found large incentive payments of £10-25 (approximately $AUD20-50) increased chlamydia testing significantly [19]. Our data suggest that a small incentive will not substantially improve chlamydia testing rates.

### Strengths and Limitations

The major strength of this study was that it was the first RCT to our knowledge to examine the effect of providing GPs with an incentive payment to increase chlamydia testing in general practice. Previous Australian studies aimed at increasing testing in general practice have examined the effectiveness of a variety of multi-faceted and singular method interventions, including the use of computer alerts [10], concurrent testing with pap smears [11] and online sexual health assessment tool referral [12]. Retention rates in the trial were very high, with a 96% response rate for testing data and questionnaire data. The study provided a good representative sample of GPs across regional/rural and metropolitan Victoria.

There were a number of limitations to our study, most notably, that the observed change in testing was considerably lower than hypothesized. An imbalanced distribution of variables was also evident at trial commencement, with GPs in the control group more likely to be younger and have a postgraduate qualification. GPs in the intervention group were also more than twice as likely to be testing for chlamydia at trial commencement. Although our multivariate analysis did adjust for these baseline differences, the lower than hypothesized effect size and the baseline imbalance in key confounders, did reduce our statistical power. The comparison of different pre-trial (12 months) and trial (6 months) time periods may not have allowed for seasonal changes in patient load and possible changes in testing frequence. In interpreting these results, it is important to note that one GP in the intervention group was testing very high numbers of young women before the trial commenced, accounting for nearly half the total number of chlamydia tests (44%) in the pre-trial period, but less than a quarter (23%) in the trial period. Despite thorough investigation into the decrease in this GP's testing figures during the trial period, no practicable explanation could be found. If this GP is removed from the multivariate analysis, a greater increase in testing in the intervention group compared to the control group is evident suggesting a positive impact of the incentive payment (from OR = 0.9; 95% CI: 0.5, 1.6 to OR = 1.22; 95% CI: 0.8, 2.0).

A further limitation of our study is that we did not provide GPs in the intervention group with ongoing feedback on their testing performance, nor were incentive payments made until the completion of the trial. It is likely that GPs forgot they were in the trial and consequently that they would receive payment for each eligible woman tested. The additional use of testing feedback on the number of tests performed during the trial period may have been useful in prompting behaviour change in GPs [29].

It is likely that testing increased in both groups due to GPs raised awareness from the educational components of the study [30-32] - both the intervention and the control group received the same educational package prior to commencement in the trial. Educational interventions as part of a multi-faceted intervention [33], and which combine strategies such as outreach visits and printed material have been shown to be effective in changing physician behaviour [30] without financial incentive. The significant decrease in positive chlamydia diagnosis in the intervention group is likely to be as a result of GPs testing higher numbers of low risk or asymptomatic women.

Lastly, female GPs and part time GPs were over-represented in the sample compared with the Australian GP population and therefore it is possible that the results of our study may not be generalizable to the wider population.

While it appears that a small financial incentive did not motivate behaviour change in GPs, large financial incentives have shown to be effective in other settings. In the UK, as part of the 1999/2000 pilot chlamydia testing program undertaken in two healthcare authorities, financial incentives of up to £25 pounds (approximately $AUD50) were offered to practices for the opportunistic chlamydia testing of young women aged 16 to 24 years. General practices comprised a high proportion of participating health sites (72%), from which an effective screening rate (ESR) of 46% in the target female population was achieved in the Portsmouth authority, where testing was fully implemented from the beginning of the program [19]. However, since the introduction of the National Chlamydia Testing Program (NCSP) in 2003, when financial incentives were discontinued, the ESR dropped significantly in general practice to around 10% [34]. Santer et al [35], in a similar study examining opportunistic chlamydia testing in primary care among women, reported an ESR of 30% in women attending for a cervical smear and 23% for contraception, however conceded it was unlikely higher rates could be achieved among teenage women (23%) without the use of a financial incentive scheme and an education campaign to raise public and professional awareness [35]. In recent Australian studies, in which GPs have been asked about how best to facilitate increased testing in general practice, the need for financial incentives has been stressed [13,15].

The size of the financial incentive offered in this study may have been insufficient to motivate GPs to offer increased testing. Systematic reviews have shown small financial incentives have mixed results in improving the quality of physician care [17,18,28]. Town et al [18], in a systematic review of the randomized trials examining the effect of financial incentives on provider preventative care (n = 8), deduced that small rewards did not appear to induce change in doctors preventative care practices. A higher incentive payment or different incentive scheme i.e. target screening rate bonuses or practice level incentives, may have proved more effective however, a cost-effectiveness analysis would need to be undertaken to compare alternative strategies to increase testing [36]. Payments in the UK pilot chlamydia testing program were considerably higher than those offered here and were offered to the practice, not the GP, however they were not sustained as part of the NCSP. In stating this, there has been evidence to suggest that small incentive payments to GPs, as part of a multi-faceted package including regular feedback, have increased childhood immunization rates in Australia [5].

The lack of reminders may have also have had a negative impact on the uptake of testing by GPs. Reminders are generally accepted as an effective way to promote behaviour change [37,38] and reviews have shown that computer based alerts can be effective in increasing GPs preventative care practices [39-41]. In a recent Australian RCT, in which intervention group GPs received a computer generated alert to remind them to test young women for chlamydia, a 27% greater increase in chlamydia testing was evident in the intervention group [10]. The authors concluded that while the computer alert increased chlamydia testing, the reminder alone would not be sufficient to increase testing to the levels required; however, it would be useful as part of a multi-faceted intervention [10].

## Conclusions

Our study suggests that a small financial incentive alone does not increase chlamydia testing in general practice. Given the limitations of the study, it is possible that small incentives with regular feedback or reminders may increase chlamydia testing in general practice; however higher financial incentives may also be required. Further research is required to determine if financial incentives can increase testing in Australian general practice, the type and level of financial scheme required and whether incentives need to be part of a multi-faceted package. A cost-effective analysis would need to be undertaken to evaluate the economic viability of alternative incentive schemes versus other possible interventions to increase chlamydia testing in general practice.

## Competing interests

This study was funded by the Victorian Department of Human Services as part of their Chlamydia Prevalence and Testing Projects. MYC and JSH were supported by NHMRC fellowships 400399 and 359276, respectively. MH received funding from the NHMRC and VicHealth during this project.

## Authors' contributions

JB was responsible for the recruitment of practices, data collection, questionnaire data analysis and preparation of the first draft of the manuscript; CF contributed to the conception, design and planning of this study and the writing, editing and approval of the manuscript; MTS contributed to the design and planning of this study, undertook the clinical audits and critically reviewed the manuscript; MP contributed to the design and planning of this study, undertook the clinical audits and critically reviewed the manuscript; KM contributed to the design and planning of this study, undertook the education sessions and QA&CPD component of the study and critically reviewed the manuscript; SB contributed to the conception, design and planning of this study and critically reviewed the manuscript; LG contributed to the conception, design and planning of this study, Stata data analysis and contributed to the writing of the manuscript; MH contributed to the conception, design and planning of this study and critically reviewed the manuscript; LS contributed to the conception, design and planning of this study and critically reviewed the manuscript; MW contributed to the conception, design and planning of this study and critically reviewed the manuscript; JW was responsible for the Stata data analysis and critically reviewed of the manuscript; MYC contributed to the conception, design and planning of this study and critically reviewed the manuscript; JH contributed to the conception, design and planning of this study, data analysis and writing, editing and approval of the manuscript. All authors read and approved the final manuscript.

## Pre-publication history

The pre-publication history for this paper can be accessed here:

http://www.biomedcentral.com/1471-2458/10/70/prepub
